# Supplementary material for: A gelatin/collagen/polycaprolactone scaffold for skin regeneration
Source: PeerJ. 2019 Feb 1;7:e6358. doi: 10.7717/peerj.6358 (PMC6361006; doi:10.7717/peerj.6358)
Supplement: Supplemental Information 7 — (Tm, melting temperature; Endo, endothermic; data H, enthalpy). [file peerj-07-6358-s009.pdf]

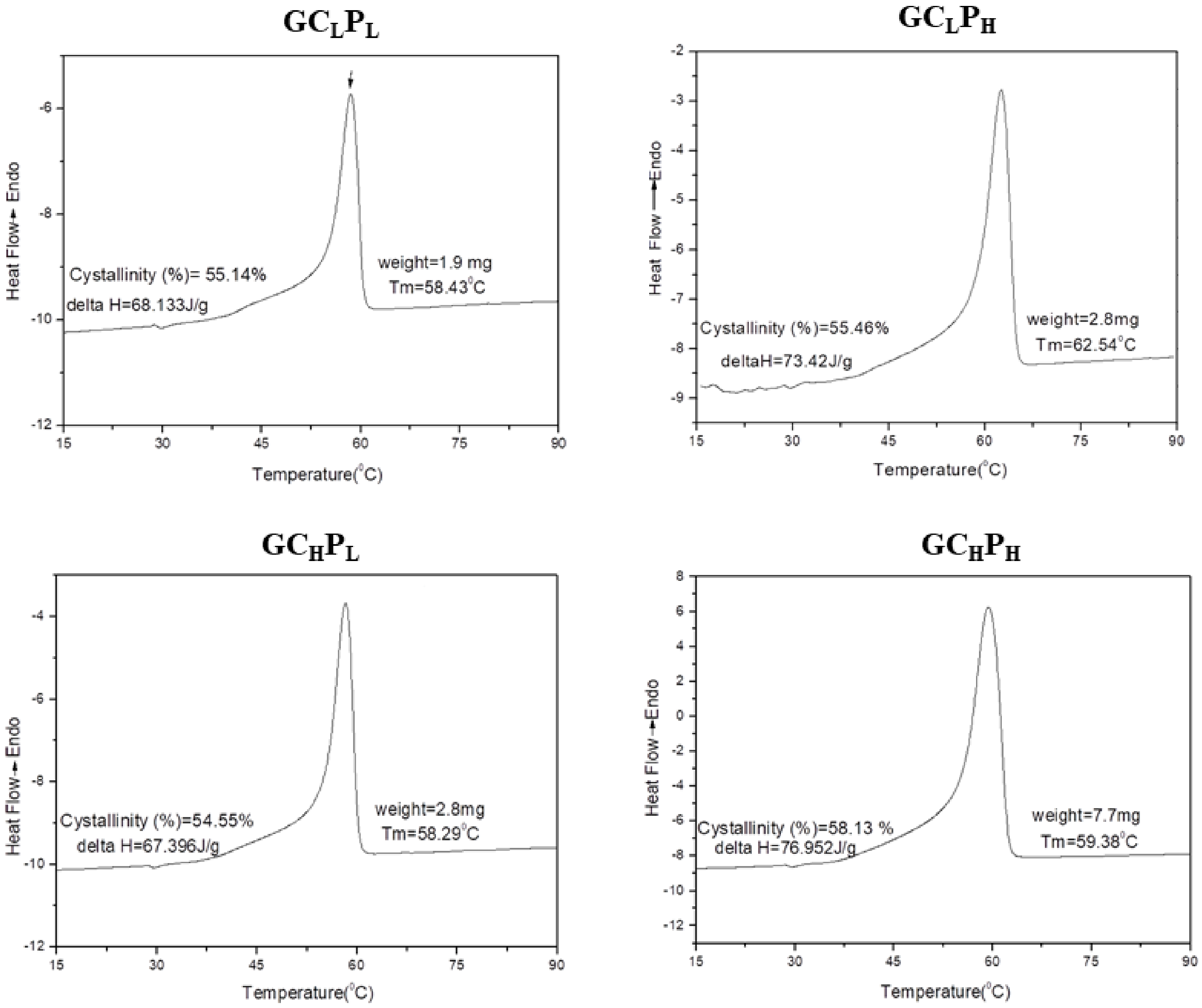

Supplement S1 Differential scanning calorimetry scan of four G/C/P biocomposites. (Tm: melting temperature; Endo: endothermic; data H: enthalpy)
